# Supplementary figures and images for: Cortical Networks Relating to Arousal Are Differentially Coupled to Neural Activity and Hemodynamics
Source: J Neurosci. 2024 May 20;44(25):e0298232024. doi: 10.1523/JNEUROSCI.0298-23.2024 (PMC11209646; doi:10.1523/JNEUROSCI.0298-23.2024)

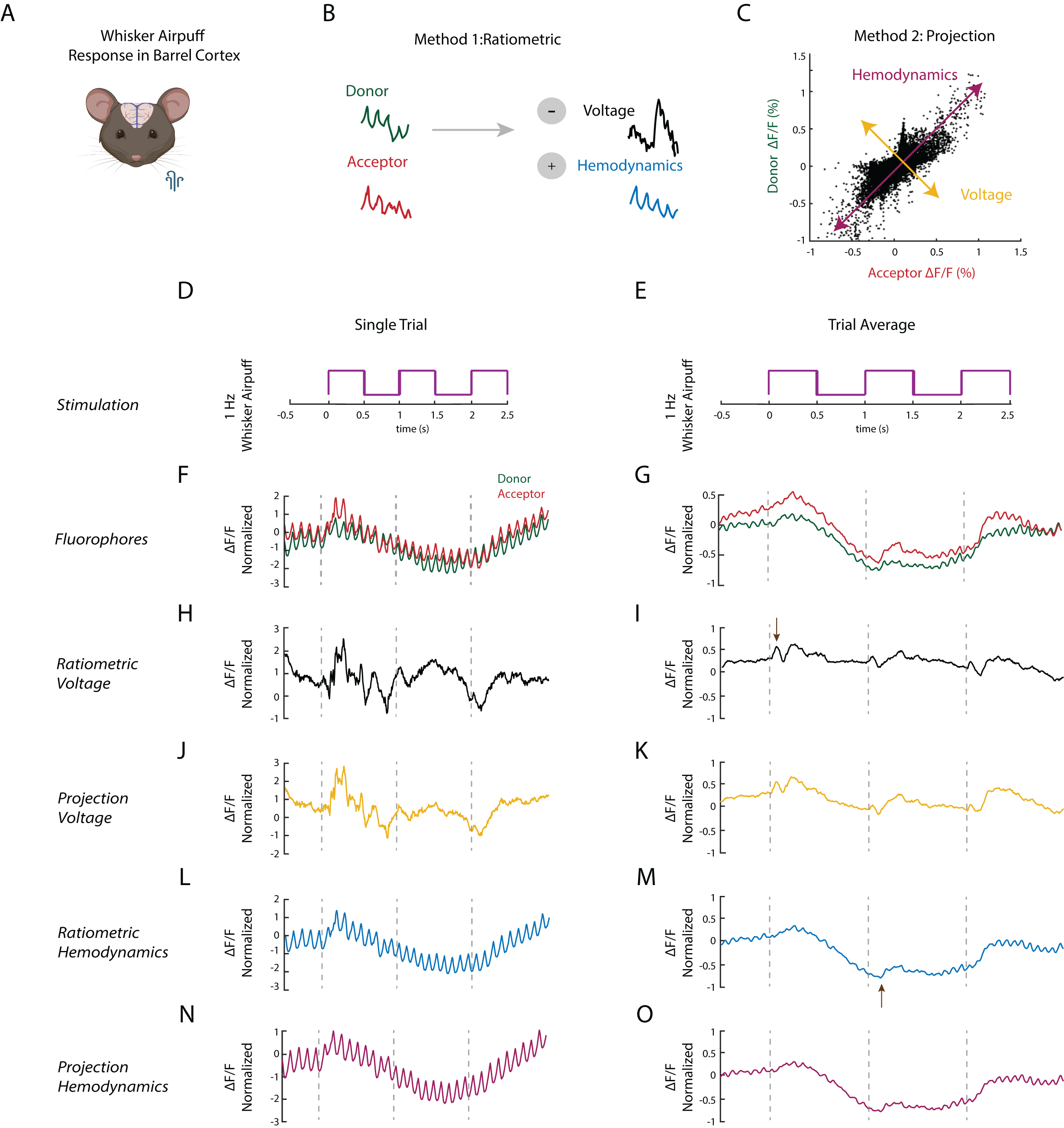

Supplement: Figure 2-1 — Comparison between ratiometric and projection method for separating voltage and hemodynamic signals A. Schematic of mouse representing air puff stimulus. B. Graphic showing how the ratiometric method takes the raw donor (green) and acceptor (red) activity and produces traces that represent voltage (black) and hemodynamic activity (blue). C. Scatter plot showing the raw acceptor and donor fluorescence values for a random selection of 1 million pixels from a representative single trial. Using the projection method one can pull out activity from two dimensions, the first representing hemodynamic activity (purple) and the second representing voltage activity (yellow). D. and E. show the time course of the 1 Hz air-puff train that was used, duty cycle 50%. F. Single trial trace of normalized changes in fluorescence for the raw donor and acceptor channel taken from an ROI over right barrel cortex. Dotted lines represent the onset of the air puff. G. same as in F but for a trial average response (n = 100 trials, from 2 mice). H. and I. The single trial and trial averaged voltage trace obtained from the ratiometric method. Arrow represents the onset of the voltage response. J. and K. The single trial and trial average voltage trace obtained using the projection method. L. and M. the single trial and trial average hemodynamic response obtained from the ratiometric version. The arrow represents the peak in changes in HbT in response to the air puff stimulation. N. and O. The single trial and trial average hemodynamic response obtained from the projection method. Download Figure 2-1, TIF file. [file jneuro-44-e0298232024-s001.tif]

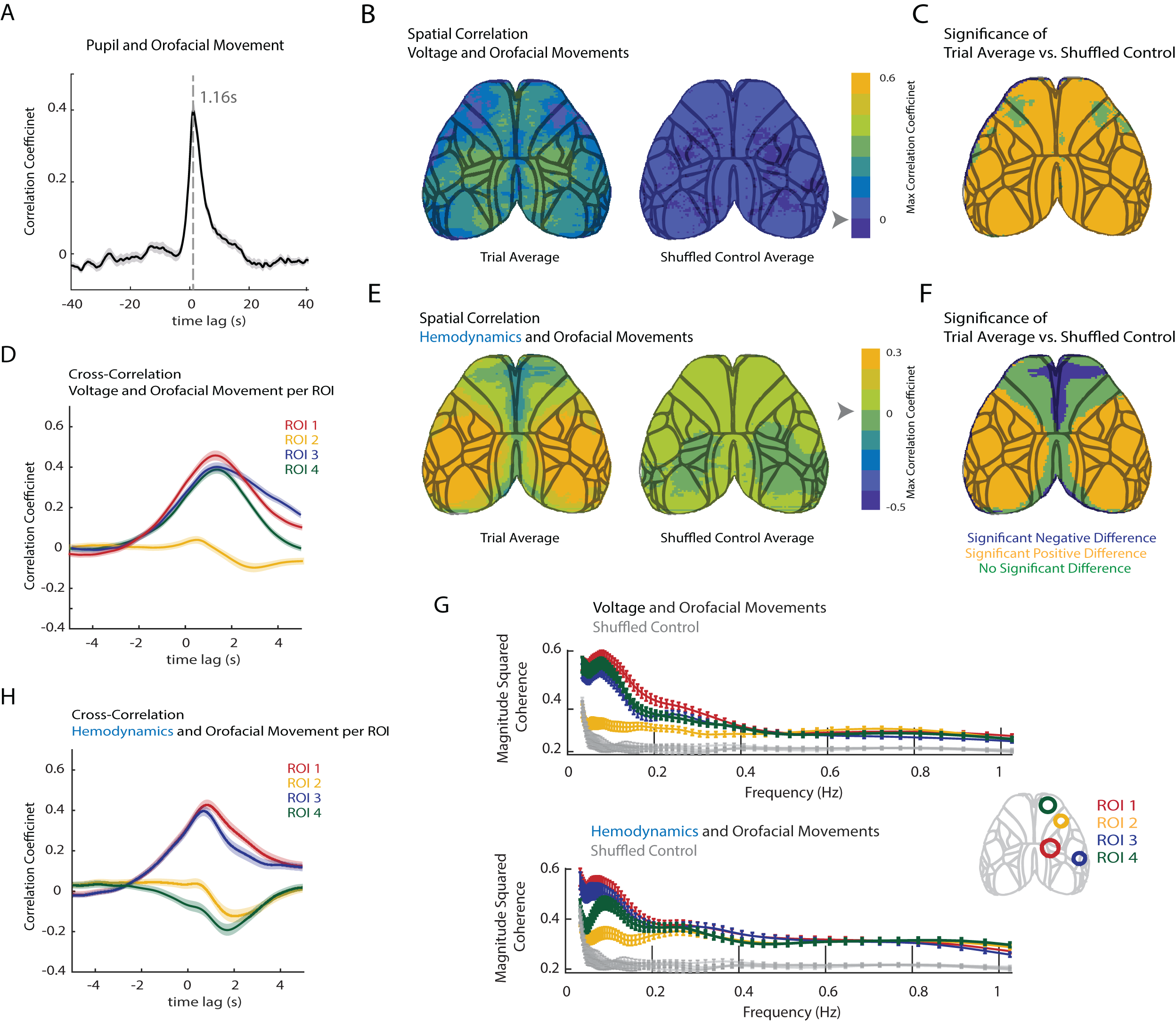

Supplement: Figure 3-1 — Region dependent coupling of orofacial movements to voltage and hemodynamic activity across functional cortical areas A. Trial average cross correlation between pupil diameter changes and orofacial movements. Max correlation of ∼0.4 at a lag of 1.16 s. B.,C.,E.,F. Same type of spatial cross correlation maps as in Figure 3 panels E,F,H,I but correlating now changes in activity to changes in orofacial movements. D., H., Same cross correlations per ROI as shown in Figure 3 panel G and J but now with relationship to changes in orofacial movements. G. Magnitude squared coherence between voltage activity and orofacial movements across 4 different ROIs. I. Same as in G but for hemodynamic activity and orofacial movements. Error bars represent standard error. For all panels that contain the hemodynamic signal, it has been inverted and lowpass filtered at 1 Hz. Download Figure 3-1, TIF file. [file jneuro-44-e0298232024-s002.tif]
